# De novo transcriptome sequencing and comparative analysis to discover genes related to floral development in *Cymbidium faberi* Rolfe

**DOI:** 10.1186/s40064-016-3089-1

**Published:** 2016-08-30

**Authors:** Yuying Sun, Guangdong Wang, Yuxia Li, Li Jiang, Yuxia Yang, Shuangxue Guan

**Affiliations:** Department of Horticulture, Nanjing Agricultural University, Nanjing, 210095 China

**Keywords:** *Cymbidium faberi* Rolfe, RNA-seq, Flower development, Flowering, Genes

## Abstract

*Cymbidium faberi* is a traditional orchid flower in China that is highly appreciated for its fragrant aroma from its zygomorphic flowers. One bottleneck of the commercial production of *C*. *faberi* is the long vegetative growth phase of the orchid and the difficulty of the regulation of its flowering time. Moreover, its flower size, shape and color are often targeting traits for orchid breeders. Understanding the molecular mechanisms of floral development in *C*. *faberi* will ultimately benefit the genetic improvement of this orchid plant. The goal of this study is to identify potential genes and regulatory networks related to the floral development in *C*. *faberi* by using transcriptome sequencing, de novo assembly and computational analyses. The vegetative and flower buds of *C*. *faberi* were sampled for such comparisons. The RNA-seq yielded about 189,300 contigs that were assembled into 172,959 unigenes. Furthermore, a total of 13,484 differentially expressed unigenes (DEGs) were identified between the vegetative and flower buds. There were 7683 down-regulated and 5801 up-regulated DEGs in the flower buds compared to those in the vegetative buds, among which 3430 and 6556 DEGs were specifically enriched in the flower or vegetative buds, respectively. A total of 173 DEGs orthologous to known genes associated with the floral organ development, floral symmetry and flowering time were identified, including 12 TCP transcription factors, 34 MADS-box genes and 28 flowering time related genes. Furthermore, expression levels of ten genes potentially involved in floral development and flowering time were verified by quantitative real-time PCR. The identified DEGs will facilitate the functional genetic studies for further understanding the flower development of *C*. *faberi*.

**Electronic supplementary material:**

The online version of this article (doi:10.1186/s40064-016-3089-1) contains supplementary material, which is available to authorized users.

## Background

Orchidaceae is one of the largest families in the angiosperms with more than 25,000 species, which displays a great biodiversity resulting from adaptation to diverse habitats (Pridgeon et al. 2005). Genomic information on orchids were mainly focused on *Phalaenopsis* (Su et al. 2011; Cai et al. 2014), *Dendrobium* (Yan et al. 2015; Zhang et al. 2016), *Cymbidium ensifolium* (Li et al. 2013), *Cymbidium sinense* (Zhang et al. 2013). *Cymbidium faberi* Rolfe., common named as “Hui Lan”, is one of the oldest and most popular orchids species cultivated in China, which is highly appreciated mainly because of its beautiful flower posture and fragrant aroma (Wolff, 1999). However, large scale commercial production of *C*. *faberi* was often hindered due to the long vegetative growth phase (usu. 5–7 years) and difficulties in flowering time control.

Plant flowering involves a transition process from vegetative growth to reproductive development with a series of conserved underlying metabolic or external phenotypic changes taking place in the shoot apical meristem. In *Arabidopsis thaliana*, there are four major pathways controlling the timing of flowering, including photoperiod, vernalization, gibberellin (GA) and autonomous pathways (Mouradov et al. 2002). Major genetic elements involved in this pathways have been defined as the key switches to control flowering, such as *CONSTANS* (*CO*) in the photoperiodic pathway and *FLOWERING LOCUS C* (*FLC*) in the autonomous and vernalization pathways (Mouradov et al. 2002). The *FLOWERING LOCUS T* (*FT*) gene activates the expression of a number of flower developmental genes (Mouradov et al. 2002). In the orchid plant *Phalaenopsis aphrodite*, *PaFT1* can suppress the delayed flowering caused by *SHORT VEGATATIVE PHASE* (*SVP*) and *FRIGIDA* (*FRI*) (Jang et al. 2015). The *Cymbidium FT* orthologous gene was also cloned and ectopic expression of *CgFT* resulted in early flowering in transgenic *Arabidopsi*s (Huang et al. 2012). Over expression of *DnAGL19*, a *SOC1*-*1*/*TM3*-like ortholog in *Arabidopsis* resulted in a slightly accelerated flowering time under normal growth conditions (Liu et al. 2016).

During the transition from vegetative growth to reproductive growth, MADS-box family genes play important roles in regulating floral organ specification, development and evolutionary in higher plants (Weigel and Meyerowitz 1994; Purugganan et al. 1995; Münster et al. 1997). Although conserved flowering pathways and multiple key genes were revealed in model plants, there is limited information of *C*. *faberi*, the very unique and important plant species featured with a 5–7 year-long vegetative growth phase. Unlike most tropical orchids (e.g. *Phalaenopsis* and *Cattleya*), *C*. *faberi* flowers are not brightly showy but of strong aromas to attract pollinating insects. It is of strong interest for orchid breeders to improve the appearance of its flower size, color and shape with uncompromised aromas. The flowers of *C*. *faberi* are zygomorphic consisting of four whorls: The first whorl is comprised of the petal-like sepals, the second whorl is of two petals and one highly specialized labellum in the middle, the third whole is of stamens, and fourth whorl is of pistils in the form a highly specialized united gynostemium (Rudall and Bateman 2002). The well-known ABC model clarify that floral development is determined by five kinds of floral organ identity genes in diverse plant groups (Coen and Meyerowitz 1991; Weigel and Meyerowitz 1994). Sepal formation is specified by the expression of A-class function genes. Expression of AB and BC determine the development of petals and stamen formation, respectively. The development of carpel is determined by C-class genes function alone, and D-class genes specify ovules. While class E function redundantly specify petals, stamens, and carpels as well as floral determinacy (Pelaz et al. 2000, 2001; Anusak et al. 2003). The floral morphology of orchid species is unique with gynostemium, labellum and resupination caused by 180° torsion of the pedicel (Rudall and Bateman 2002). ‘The Perianth (P) code’ clarifies the mechanisms of sepal/petal/lip determination in *Oncidium* and *Phalaenopsis* orchids. The competition between different *APETALA3*/*AGAMOUS*-*LIKE6* (*AP3/AGL6*) homologues determines the formation of the complex perianth patterns in orchids. The formation of sepal/petal were specified by the higher-order heterotetrameric SP (sepal/petal) complex (*OAP3*-*1*/*OAGL6*-*1*/*OAGL6*-*1*/*OPI*), whereas the lip formation required the L (lip) complex (*OAP3*-*2*/*OAGL6*-*2*/*OAGL6*-*2*/*OPI*) exclusively (Hsu et al. 2015). Other MADS-box function genes participating in the sepal and petal development were also isolated from *Dendrobium madame*, *D*. *crumenatum*, *Oncidium* Gower Ramsey (Yu et al. 2000, 2002; Hsu and Yang 2002; Hsu et al. 2003; Xu et al. 2006). Genes involved in flower identity and floral organ specification are still unknown in *C*. *faberi* yet.

Besides petal shape and size, floral symmetry significantly affects the ornamental value of flowers. So far, three transcription factors (TFs) that determine the floral symmetry are identified, including *CYCLOIDEA* (*CYC*) from the TCP family (*TEOSINTE BRANCHED1/CYCLOIDEA/PROLIFERATING CELL NUCLEAR ANTIGEN FACTOR*), *DIVARICATA* (*DIV*) and *RADIALIS* (*RAD*) from the MYB family (Luo et al. 1996; Doebley et al. 1997; Almeida et al. 1997; Galego and Almeida 2002; Corley et al. 2005; Costa et al. 2005). Of these genes, *CYC* and its orthologues establish the floral monosymmetry through specifying dorsal flower identity (Luo et al. 1996, 1999; Feng et al. 2006; Wang et al. 2008). Studies on floral symmetry genes mainly focused on species with highly derived morphologies in eudicots, whereas only a few studies are available for monocots (Luo et al. 1996, 1999; Feng et al. 2006; Wang et al. 2008; Yuan et al. 2009; Bartlett and Specht 2011; Preston and Hileman 2012; Hoshino et al. 2014). Orchidaceae is characterized by highly specialized zygomorphic flowers. Studies on orchid flower symmetry is very limited (Paolo et al. 2015). Overall, the detailed molecular mechanism or the genetic elements in the regulation of floral organ specification and development in *C*. *faberi* remains elusive.

Transcriptome analysis is an useful tool to discriminate differences in transcript abundance among different cultivars, organs and different treatment conditions in model and non-model plants (Cheung et al. 2006; Trick et al. 2009; Li et al. 2013; Hyun et al. 2014; Zhang et al. 2014). In order to excavate genes that might regulate the floral development in *C*. *faberi*, we used high-throughput Illumina sequencing and bioinformatics analysis to compare the de novo transcriptomes of vegetative and flower buds from *C*. *faberi*. The vegetative transcriptome can be used to find genes related to the vegetative growth. The floral transcriptome was sufficiently comprehensive for gene discovery and analysis of major metabolic pathways associated with flower traits. Genes expressed differently in the vegetative buds and flower buds may play important roles in the vegetative growth or floral development. Through transcriptome analysis, large numbers of genes related to floral organ initiation, flower symmetry patterning and flowering were identified in *C*. *faberi*. These results provide fundamental information for further studies on the molecular mechanism of flower development in *C*. *faberi*.

## Methods

### Plant materials

**Fig. 1:**
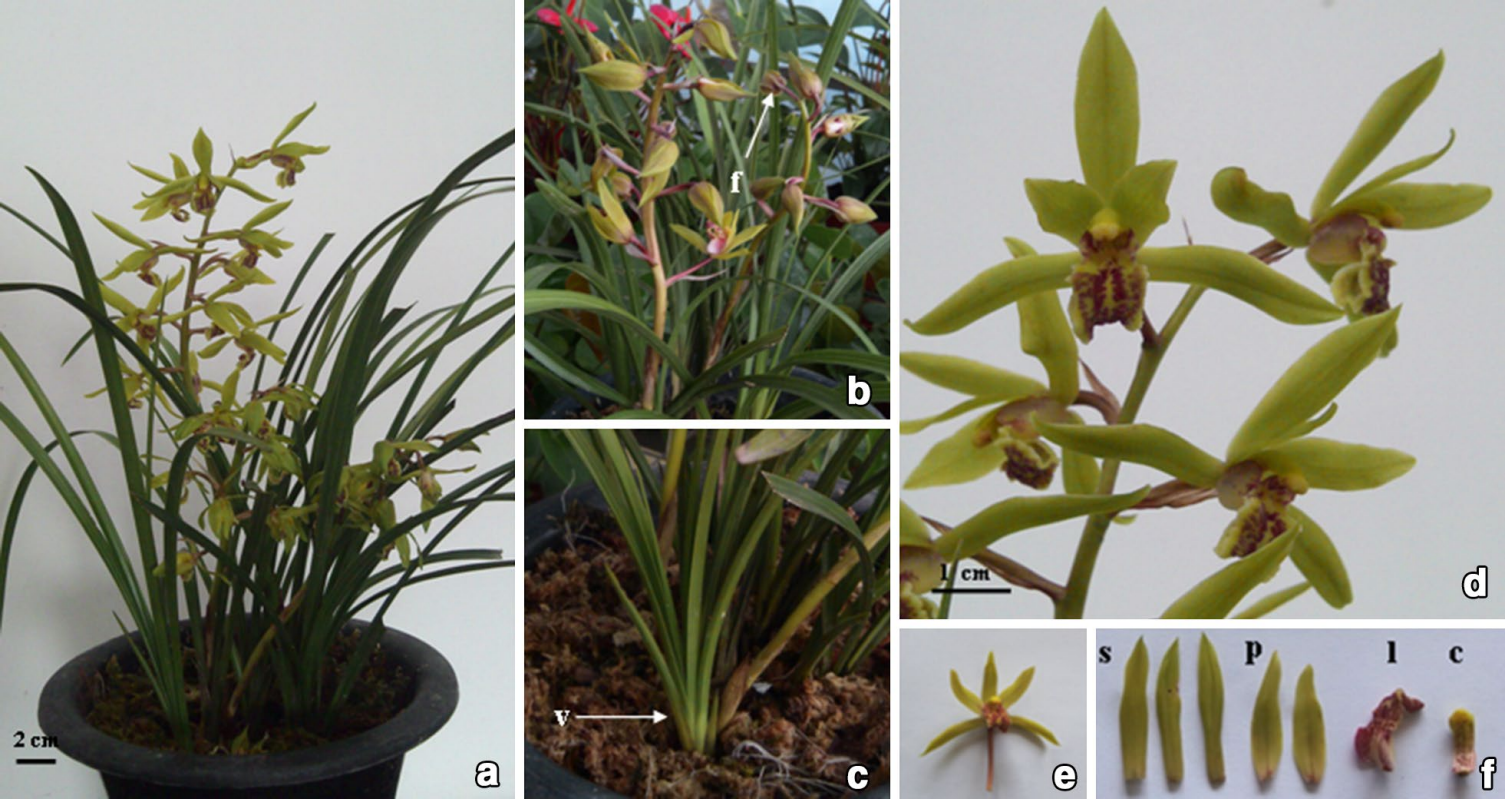
The mature plants and flowers of *C*. *faberi*. **a** Flowering plants of *C*. *faberi*; **b** Inflorescence with flower buds (*f*); **c** Vegetative buds (*v*); **d** blooming flowerers; **e** single flower; **f** different parts of a single flower, *s* sepal; *p* petal; *l* labellum; *c* column

Mature plants of *C*. *faberi* with light green flowers, originally introduced from Henan province of China, were grown in the greenhouse at Department of Horticulture, Nanjing Agricultural University (Nanjing, China) under natural light and a controlled temperature of 22–28 °C. The vegetative buds were collected from lateral buds, and the flower buds (0.5–1.0 cm in length) were collected from the peduncle (Fig. 1). Organs from three individual plants were pooled as one sample. The fresh samples were frozen immediately in liquid nitrogen and stored at −80 °C.

### RNA extraction, cDNA library construction and de novo assembly

Total RNA of flower and vegetative buds were extracted using EASYspin plant RNA rapid extraction kit according to the manufacturer’s protocol (Yuan Ping Hao Biotechnology Co. Ltd, Beijing, China). Then, the concentration was detected by a NanoDrop 2000™ micro-volume spectrophotometer (Thermo Scientific, Waltham, MA, USA) and the quality was tested by gel electrophoresis. cDNA library construction for the flower and vegetative buds and Illumina deep sequencing were performed on the HiSeq™ 2000 platform according to the manufacturer’s instructions at Hangzhou Woosen Biotechnology Co. Ltd. (Hangzhou, Zhejiang, China). The Illumina reads were assembled to obtain the contigs and unigenes using Trinity software and Cap3 after removing the short raw reads and quality inspection by fastQC (Grabherr et al. 2011).

### Functional annotation of unigenes

The unigenes were annotated with the National Center for Biotechnology Information non-redundant databases (NR), Gene Ontology (GO), Clusters of Orthologous Groups (COG) and Kyoto Encyclopedia of Genes and Genomes (KEGG) databases using BLASTX searches (E-value ≤ 1.0e^−5^). The Blast2GO software package and WEGO software were employed to compare and determine unigenes’ GO annotations and obtain GO functional classifications for all annotated unigenes (Götz et al. 2008; Ye et al. 2006).

### Identification of differentially expressed genes (DEGs)

Expression levels of all unigenes were calculated and then compared between the two tissue samples using the fragments per kilobase of transcript per million reads of library method (FPKM). The false discovery rate (FDR) was adopted to determine the threshold of P-values in multiple tests. DEGs were also annotated with GO assignments, COG assignments and KEGG pathways. The criteria FDR < 0.05 was used to identify DEGs and acted as a threshold of significant difference of gene expression in GO terms, COG classification and KEGG annotation.

### Quantitative real-time PCR (qRT-PCR) analysis

Total RNA of flower buds and vegetative buds were extracted as above and the first strand cDNA was synthesized using PrimeScript RT reagent Kit With gDNA Eraser (Takara Bio Inc.). All primers used in this study were designed by the Primer 5 software according to the RNA-Seq data. *CfGAPDH* (JX560732) was selected as an internal reference (Additional file 1: Table S1).

The qRT-PCR analysis was performed on ABI 7500 Real-Time PCR Detection System (Applied Biosystems) using the SYBR^®^ Premix ExTagTM reagent kit (Takara Bio Inc.) according to the manufacturer’s protocol. The PCR reactions were 40 cycles (95 °C for 15 s; 55 °C for 15 s; 72 °C for 20 s) according to the instruction manual. A melting curve was generated to test the specificity of products after the qRT-PCR. The relative expression levels of the selected unigenes were normalized to *CfGAPDH* gene and calculated using the 2^−ΔΔCt^ method. Data were derived from three independent replicates.

## Results

### Transcriptome sequencing and de novo assembly

Equal amount of RNAs from the vegetative and flower buds were used to constructed cDNA libraries separately, and then sequenced. A total of 35,511,583 and 32,514,423 raw reads were obtained in the flower and vegetative buds, respectively. After removing the short raw reads and quality inspection by fastQC, the RNA-seq produced 35,510,239 and 32,513,288 clean reads for the flower and vegetative buds, respectively (Table 1). All of these reads were employed for further de novo assembly. And 189,300 contigs with an average length of 755 bp were generated using the Trinity software package. These contigs were assembled into 172,959 unigenes (200 to >10,000 bp) with an average length of 698 bp and an N50 (N50 represents weighted median length of all contigs) of 1340 bp (Table 2). Among them, 59,505 (34.4 %) unigenes were more than 500 bp. The size distribution of the assembled unigenes is shown in Additional file 2: Fig. S1. And this transcriptome shotgun assembly project has been deposited at DDBJ/EMBL/GenBank under the accession of GDHD00000000.

**Table 1:** Statistical summary of *C. faberi* transcriptome sequencing data

Statistics of data production	Flower buds	Vegetative buds
Raw reads	35,511,583	32,514,423
Raw bases	5,326,737,450	4,877,163,450
Clean reads	35,510,239	32,513,288
Clean bases	5,280,378,528	4,841,503,927
Clean data rate (%)	99.125	99.27

**Table 2:** Summary of assembly quality from transcriptome in *C. faberi*

	Contigs	Unigenes
Total number	189,300	172,959
Average length(bp)	755.549	698.016
Max length(bp)	11,797	11,797
Min length(bp)	201	201
GC percentage (%)	42.1	42.0
Length of N50 (bp)	1452	1340

### Functional annotation of *C*. *faberi* transcriptome

To validate and annotate the assembled unigenes, the 172,959 unigenes generated were subjected to BLASTX searches (E-value ≤ 1.0e^−5^) against public protein databases such as NCBI NR, GO, COG and KEGG. As a result, a total of 65,577 (37.91 %) unigenes were predicted to be coding sequence and 66,000 (38.16 %), 50,161 (29.00 %), 27,443 (15.87 %), 19,715 (11.40 %) unigenes had homologous sequences respectively in NR, GO, COG and KEGG databases (Table 3).

**Table 3:** Summary statistics of functional annotation for *C. faberi* vegetative and flower buds unigenes in public databases

Public protein database	No. of unigene hits	Percentage (%)
Predict protein number	65,577	37.91
COG	27,443	15.87
NR (E-value < 10^−5^)	66,000	38.16
GO	50,161	29.00
KEGG	19,715	11.40

**Fig. 2:**
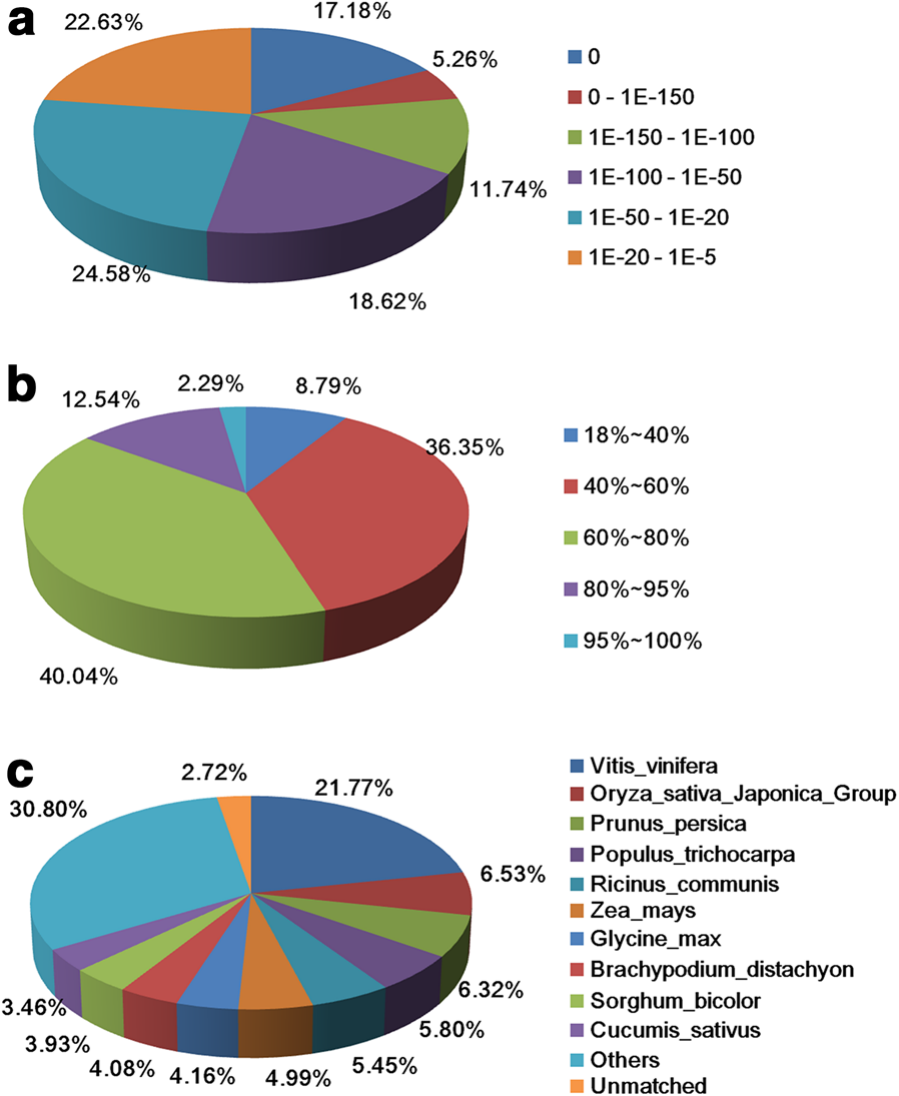
Characteristics of sequence homology of *C*. *faberi* BLASTED against NCBI non-redundant (Nr) database. **a** E-value distribution of BLAST hits for matched unigene sequences, using an E-value cutoff of 1.0E^−5^. **b** Similarity distribution of top BLAST hits for each unigene. **c** Species distribution of the top BLAST hits

According to the NR annotation, 66,000 (38.16 %) unigenes had homologous sequence in the database, of which 14,365 (21.77 %), 4309 (6.53 %), 4173 (6.32 %) unigenes were annotated with homologous genes from *Vitis vinifera*, *Oryza sativa* Japonica Group and *Prunus persica*, respectively (Fig. 2a). The similarity distribution indicated that 54.87 % of the unigenes showed a similarity higher than 60, and 40.04 % unigenes had similarity between 60 and 80 % (Fig. 2b). For the E value distribution, 53.79 % of the top hits had high homology with the E-value <1.0e^−50^ (Fig. 2c).

**Fig. 3:**
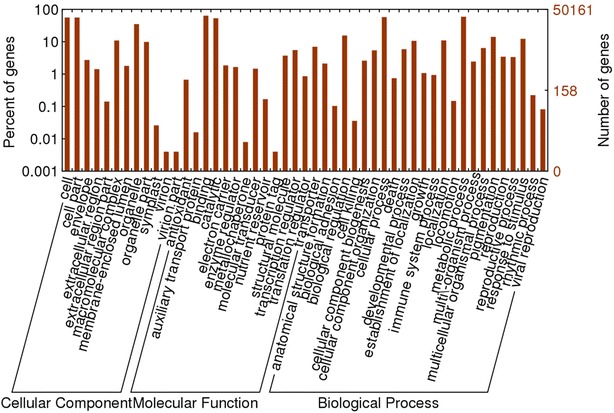
GO classification of all annotated unigenes. GO classification of all annotated 50,161 unigenes. All terms belonged to the three main GO categories: biological process, cellular component and molecular function. The *x*-axis indicated the subcategories, the right *y*-axis indicated the number of genes in each category, the *left y*-axis indicated the percentage of a specific category of genes in the corresponding GO category. *Red column* indicated all annotated unigenes

As an international standardized gene functional classification system, GO describes properties of genes and their products in many organisms and provides a comprehensive description of gene properties across species and databases (Hao et al. 2011). Based on the BlastX results, a total of 50,161 (29.00 %) unigenes were assigned into 49 GO term annotations, including three categories, i.e., molecular function, biological process, and cellular component (Fig. 3). Within the molecular function category (70,280 GO terms), “binding” (32,021, 45.6 %) and “catalytic activity” (26,742, 38.0 %) were the most highly represented terms. For biological process (108,214 GO terms), “cellular process” (28,941, 26.7 %) and “metabolic process” (29,813, 27.6 %) were the highly represented terms. Among the cellular component category (87,253 GO terms), “cell” (28,076, 32.2 %), “cell part” (13,723, 15.7 %) and “organelle” (28,076, 32.2 %) were the three main categories.

**Fig. 4:**
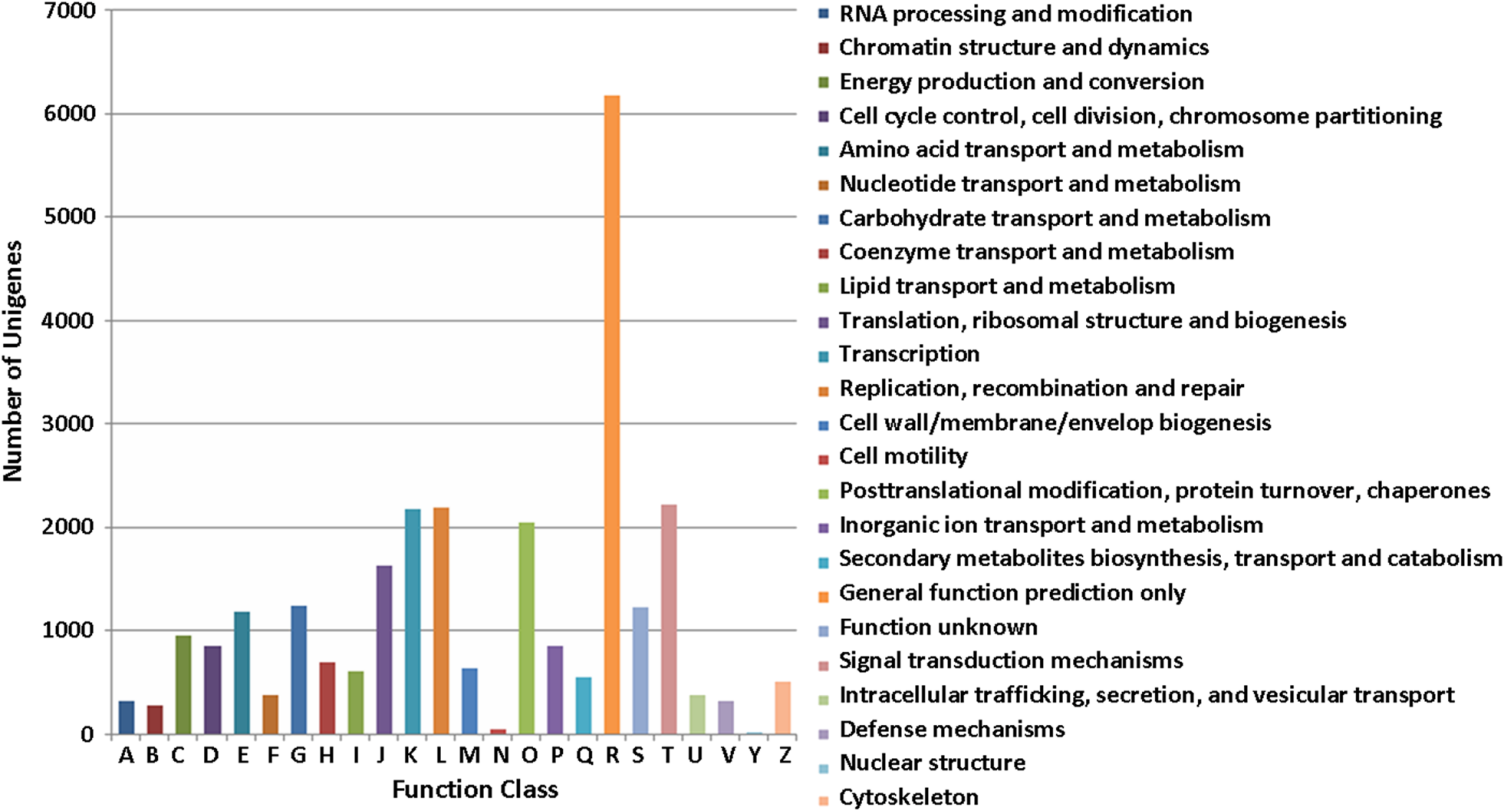
COG annotations of putative proteins. All putative proteins were aligned to the COG database and were functionally classified into at least 24 molecular families. The capital letters in *x*-axis indicates the COG categories as listed on the right of the histogram and the *y*-axis indicates the number of unigenes in the corresponding COG category

A total of 27,443 unigenes were classified into 24 COG functional categories (Fig. 4). And the largest category was “general function prediction only” (6177, 22.5 %), followed by “signal transduction mechanisms” (2220, 8.1 %) and “replication, recombination and repair” (2196, 8.0 %). Unigenes annotated as the “signal transduction mechanisms” in our study may allow for the identification of novel genes involved in signal transduction pathways. Approximately 16.9 % of the unigenes were associated with biochemical synthesis and metabolism, such as “carbohydrate transport and metabolism” (1242, 4.5 %), “amino acid transport and metabolism” (1184, 4.3 %) and “secondary metabolites biosynthesis, transport and catabolism” (544, 2.0 %). A total of 1225 unigenes (4.46 %) was “function unknown”.

Furthermore, we classified 19,715 (11.40 %) unigenes into 274 KEGG pathways by searching against the KEGG database (Additional file 3: Table S2). Among these pathways, “metabolic pathways”(3684, 18.7 %) represented the largest group, followed by “biosynthesis of secondary metabolites”(1616, 8.2 %). The other highly represented pathways included “microbial metabolism in diverse environment (803, 4.1 %)”, “ribosome (509, 2.6 %)” and “protein processing in endoplasmic reticulum (507, 2.6 %)” (Table 4). A total of 301 unigenes involved in plant hormone signal transduction were found, including 9 pathways controlling the signal transduction of auxin, cytokinin, gibberellin acid, abscisic acid, ethylene, brassinosteroid, jasmonic acid and salicylic acid (Table 5). Besides, through the KEGG pathway annotation, a circadian rhythm pathway including 58 unigenes was also found, which can be used for further studies on the flowering-related genes of the photoperiod pathway.

**Table 4:** Significantly enriched pathways of all unigenes and DEGs in *C. faberi*

Pathway	No. of all genes with pathway annotation (19,715)	No. of DEGs with pathway annotation (66)		Pathway ID
		Up-regulated genes	Down-regulat-ed genes	
Metabolic pathways	3681	60	240	ko01100
Biosynthesis of secondary metabolites	1616	66	186	ko01110
Microbial metabolism in diverse environments	803	46	96	ko01120
Ribosome	509	18	71	ko03010
Protein processing in endoplasmic reticulum	507	14	48	ko04141
Spliceosome	469	27	17	ko03040
RNA transport	457	21	21	ko03013
Starch and sucrose metabolism	403	12	48	ko00500
Cell cycle	355	25	8	ko04110
Tuberculosis	337	11	26	ko05152
Pyrimidine metabolism	330	19	13	ko00240
RNA degradation	327	19	7	ko03018

**Table 5:** The pathways involved in plant hormone biosynthesis

Pathway	Product	Pathway ID	Unigene number
Cysteine and methionine metabolism	Ethylene	ko00270	125
Phenylalanine metabolism	Salicylic acid	ko00360	103
Tryptophan metabolism	Auxin	ko00380	90
alpha-Linolenic acid metabolism	Jasmonic acid	ko00592	59
Diterpenoid biosynthesis	Gibberellin	ko00904	47
Carotenoid biosynthesis	Abscisic acid	ko00906	28
Zeatin biosynthesis	Cytokinin	ko00908	18
Brassinosteroid biosynthesis	Brassinosteroid	ko00905	17
Indole alkaloid biosynthesis	Indole-acetic acid	ko00901	2

Based on the annotation of unigenes, 118 MADS-box genes were also discovered (Additional file 4: Table S3), these including the sepal development related genes such as *APETALA1* (*AP1*), *CAULOFLOWER* (*CAL*); petal development related genes *DEFICIENS* (*DEF*)and *PISTILLATA* (*PI*); the C/D/E class function genes *AGAMOUS* (*AG*), *SEEDSTICK* (*STK*) and *FLORAL BINDING PROTEIN*-LIKE (*FBP*-like) were also identified. TCP gene family play important role in the development of plants and can be divided into two classes: PCF class and TCP-C class (which involes the CYC/TB1 clade and CIN clade) (Howarth and Donoghue 2006; Navaud et al. 2007). The CYC/TB1 clade includes genes mainly involved in the development of axillary meristems and floral bilateral symmetry (Luo et al. 1996; Doebley et al. 1997). To determine the TCP gene family in *C*. *faberi*, we analyzed the transcriptome database generated by this study and found 35 unigenes annotated as TCP TFs, including 14 *CIN*-like genes, 20 *PCF*-like genes and 1 *CYC*/*TB1*-like genes. As shown in Additional file 5: Table S4, they showed homology with 13 *Arabidopsis* TCP genes.

A total of 240 genes associated with flowering time were obtained (Additional file 6: Table S5). These include floral meristem identity genes *LEAFY* (*LFY*) and *APETALA1* (*AP1*); autonomous pathways genes *FCA*, *FPA*, *FLOWERING LOCUS* (*FLD*), *FY*, *FVE*, *FLOWERING LATE KH MOTIF* (*FLK*); vernalization pathways genes *FRI* and *VERNALIZATION INSENSITIVE* (*VIN*); photoperiod pathway genes such as *FT*, *Phytochrome A* (*PHYA*), *Phytochrome B* (*PHYB*), *PIF3*, *ELF3*, *LHY*, *SUPPRESSOR OF OVEREXPRESSION OF CONSTANS 1* (*SOC1*), *CIRCADIAN CLOCK ASSOCIATED1* (*CCA1*) and *CO*; Gibberellin (GA) pathway genes such as *GIBBERELLIC ACID INSENSITIVE* (*GAI*). All these unigenes provide important resources for future study of floral organ development, floral bilateral symmetry and flowering time.

### DEG analysis of vegetative and flower buds

**Fig. 5:**
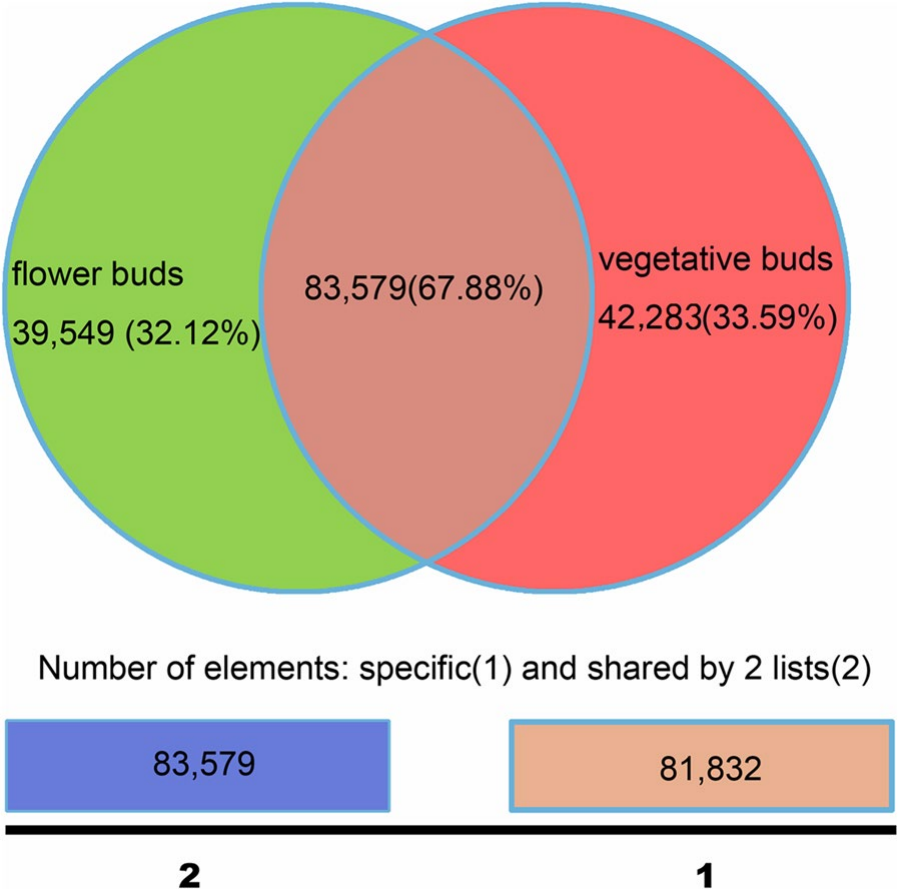
The numbers of specific genes and shared genes between the flower and vegetative buds of *C*. *faberi*

**Fig. 6:**
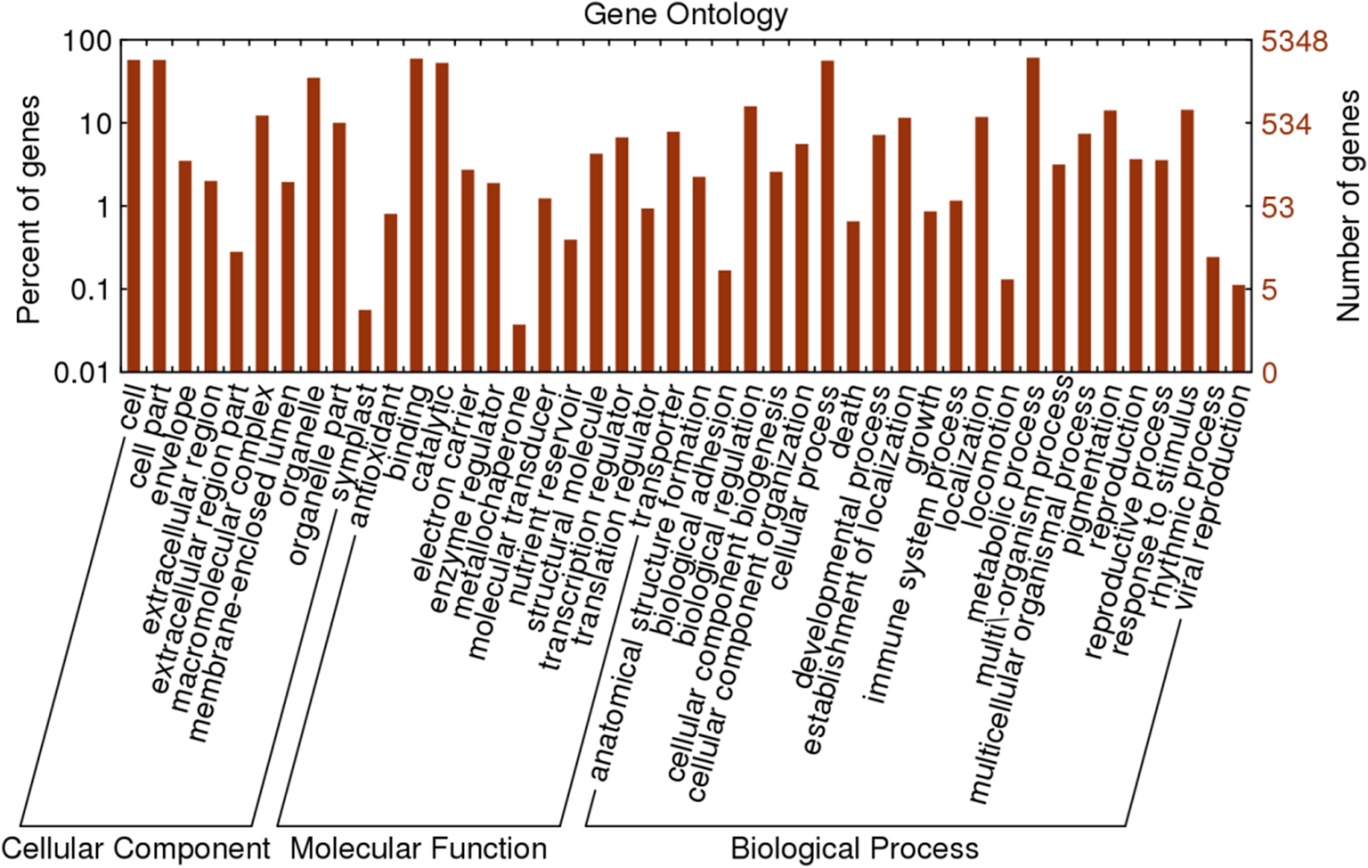
GO classification of DEGs

**Fig. 7:**
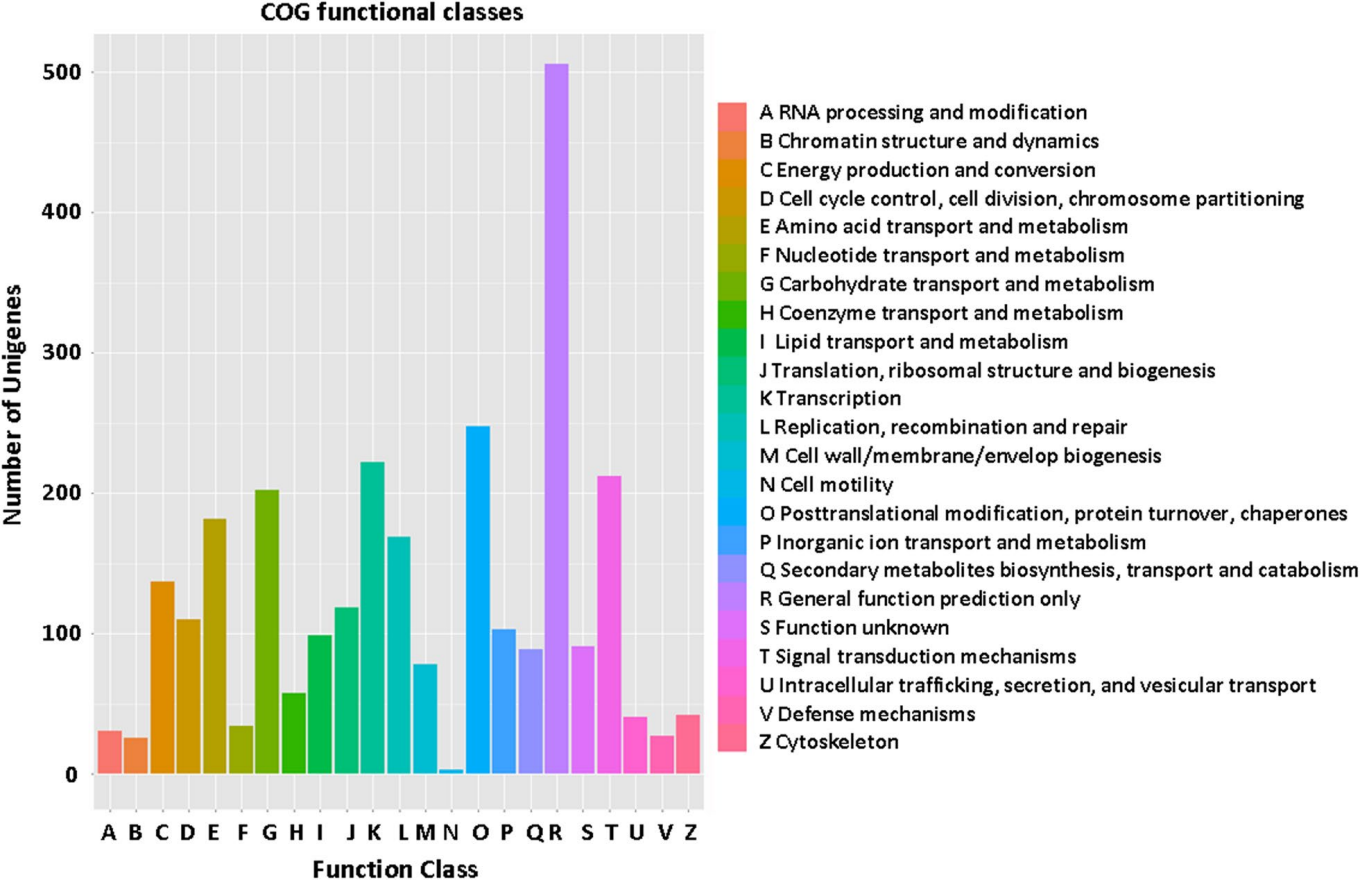
COG annotations of DEGs. 2829 DEGs were aligned to the COG database and were functionally classified into at least 23 molecular families. The *capital letters* in *x*-axis indicates the COG categories as listed on the *right of the histogram* and the *y*-axis indicates the number of unigenes in the corresponding COG category

The FPKM methods were used to analyze the gene expression in the two libraries: 123,128 and 125,862 unigenes were obtained in the flower buds and vegetative buds, of which 39,549 and 42,283 genes expressed specifically in the flower buds and vegetative buds, respectively (Fig. 5). To analyze different gene expression in the two libraries, 13,484 DEGs were identified using FPKM methods. Among them, 7683 were down-regulated and 5801 were up-regulated in the flower buds when compared to those in the vegetative buds, including 3430 and 6556 genes specifically enriched in flower and vegetative buds, respectively. To validate and annotate the assembled DEGs, the 13,484 DEGs were subjected to BLASTX comparisons (*E*-value ≤10^−5^) against GO, COG and KEGG database to identify putative functions of these unigenes. As a result, 5348 (39.7 %), 2829 (21.0 %) and 3927 (29.1 %) DEGs had homologous sequences in GO, COG and KEGG databases, respectively. In addition, 5348 DEGs were classified into 44 GO term annotations, including cellular component (1399), biological process (3921) and molecular function (7006), with multiple terms assigned to the same transcript (Fig. 6). A total of 2829 DEGs were classified into 23 functional COG categories (Fig. 7). “general function prediction only” (506) was the most, followed by “posttranslational modification, protein turnover, chaperones” (248) and “transcription” (222). To further understand the function in biological processes, 3927 DEGs were classified into 245 KEGG pathways. These results showed that most of the DEGs were involved in the “metabolic pathways”(300), “biosynthesis of secondary metabolites”(252), “microbial metabolism in diverse environment (142)” and “ribosome”(89) (Table 4). After transcriptome analysis, TFs involved in the floral differentiation were revealed and their expression levels in the flower and vegetative buds were calculated and compared using the FPKM method.

A total of 173 DEGs related to the floral development were discovered in our transcriptome data (Additional file 7: Table S6). These TFs were attributed to different gene families, including MADS (34 DEGs), TCP (12 DEGs), MYB (34 DEGs), ARF (9 DEGs), NAC (21 DEGs), b-ZIP (17 DEGs), WRKY (18 DEGs) (Table 6). Twenty-eight DEGs related to the flowering time were identified (Additional file 8: Table S7) and some shown in Table 7. Most of these TFs showed higher expression in the flower buds indicating that these genes might involve in the flower development.

**Table 6:** DEGs related to floral differentiation in the flower and vegetative buds

Genes related to floral differentiation	Total No. of DEGs	No. of DEGs in flower buds	No. of up-regulated DEGs in flower buds	No. of DEGs in vegetative buds	No. of up-regulated DEGs in vegetative buds
MADS	34	33	29	8	5
TCP	12	7	0	12	12
MYB	34	30	29	11	5
ARF	9	7	1	9	8
NAC	21	20	20	8	1
b-ZIP	17	14	13	13	4
WRKY	18	17	15	10	3

**Table 7:** Some DEGs related to flowering time in the flower and vegetative buds

Genes related to flowering	Total No. of DEGs	No. of DEGs in flower buds	No. of up-regulated DEGs in flower buds	No. of DEGs in vegetative buds	No. of up-regulated DEGs in vegetative buds
PHYA	1	1	0	1	1
CCA1	3	2	2	3	1
LHY	1	1	1	1	0
FCA	2	2	1	1	1
FY	1	1	0	1	1
FRI	3	1	1	2	2
CO	2	1	1	2	1

### qRT-PCR validation of selected genes

**Fig. 8:**
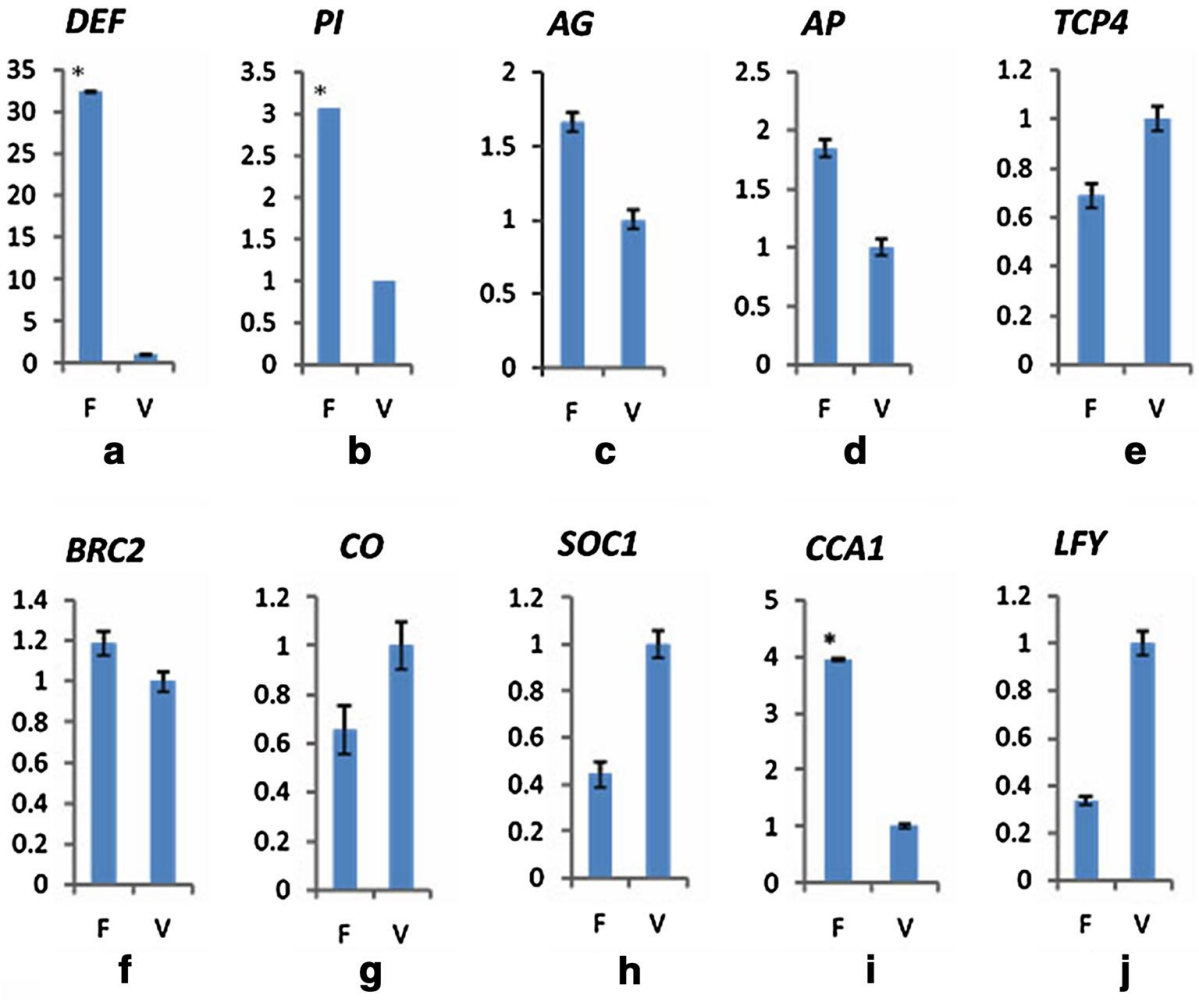
qRT-PCR analysis of ten selected genes in different organs of *C*. *faberi.* **a**–**j** indicated the relative expression level of *DEF*, *PI*, *AG*, *AP*, *TCP4*, *BRC2*, *CO*, *SOC1*, *CCA1*, *LFY* gene, respectively. In these pictures, *F* flower buds; *V* vegetative buds; *Asterisk* indicated significantly higher expression

To verify the reliability of RNA-Seq data and explicit the expression patterns of genes related to floral development, ten genes mainly related to the floral organ development, floral symmetry and flowering time associated genes were selected to perform qRT-PCR analysis. Our results indicated that *DEF*, *PI*, *AG*, *AP1*, *BRANCHED2* (*BRC2*) and *CCA1* had higher expression and *TCP4*, *CO*, *SOC1* and *LFY* displayed lower expression in the flower buds (Fig. 8a-j ). *PI* and *DEF*, the two B class function genes controlling the petal development, showed significantly higher expression in the flower buds than the vegetative buds. While *CCA1* participating in the circadian rhythm showed significantly higher expression in the flower buds than the vegetative buds. Overall, all of the 10 genes performed the same expression patterns in our qRT-PCR results as in the RNA-Seq data, verifying the reliability of the Illumina sequencing data.

## Discussion

### Capacity of the transcriptome database

In recent years, transcriptome analysis based on deep RNA sequencing has been applied to many plants to identify differences in gene expression levels among different cultivars, organs and different treatment conditions (Chen et al. 2014; Yates et al. 2014). In this study, we obtained 189,300 contigs and identified 172,959 unigenes by de novo assembly through RNA-Seq technology in the vegetative and flower buds of *C*. *faberi*. According to the Nr protein database, 66,000 (38.16 %) unigenes were successfully annotated. Furthermore, 173 DEGs involved in floral organ development, floral zygomorphy and flowering time were found. These results supported that plant conservative genes, *C*. *faberi*-specific genes, and *C*. *faberi* tissue-specific genes all were identified in our transcriptomic analysis. Our research will provide valuable information for future study to inquire the flower development mechanisms of *C*. *faberi*.

### Genes related to the floral organ development

MADS-box genes are known for their roles in the flower organ development in *Arabidopsis* and *Antirrhinum* (Coen and Meyerowitz, 1991; Weigel and Meyerowitz, 1994). While some monocot species like the orchid family possess distinct floral structures, thus floral patterning in *Arabidopsis* may not be comparable to such flowering plants. In recent years, a few MADS-box genes have been identified and characterized in *D*. *thyrsiflorum* (Reichb. f.) (Martin et al. 2006), *Oncidium* Gower Ramsey (Hsu and Yang 2002), *Phalaenopsis* (Tsai et al. 2004) and *C*. *faberi* (Xiang et al., 2011). According to the “Orchid code” theory, the identity of the lateral petals and floral lip were determined by four different *AP3/DEF*-like genes, whereas the *PI*/*GLO*-like genes retained the function unchanged like class A, C, D and E genes (Mondragón-Palomino and Theissen 2008, 2009, 2011; Aceto and Gaudio 2011). In this study, 34 DEGs of MADS-like genes were found. Meanwhile, 29 DEGs were up-regulated in the flower buds (26 DEGs specifically expressed in the flower buds and most of them were class B and class C genes) and only 5 DEGs were up-regulated in the vegetative buds. Meanwhile, the expression level of *PI* and *DEF* showed significantly higher in the flower buds than the vegetative buds, proving that *PI* and *DEF* played a pivotal role in the flower development.

### Genes related to the floral zygomorphy

The ornamental value of *C*. *faberi* is determined by many factors, especially the novel flower color, shape and fragrance. Floral zygomorphy have evolved multiple times from radially symmetrical (actinomorphic; polysymmetric) ancestors in different angiosperm lineages (Endress 1999; Stebbins 1974). The mechanism of TCP model for bilateral flower symmetry has been well established in model species, such as snapdragon (*A*. *majus*), *Lotus japonicus* and rice (Luo et al. 1996; Feng et al. 2006; Yuan et al. 2009). In *A*. *majus*, *CYC* and *DICH* control the dorsoventral asymmetry (Luo et al. 1999). In *L*. *japonicus*, floral dorsoventral asymmetry is regulated by three *LjCYC* genes (*LjCYC*1, *LjCYC*2, *LjCYC*3), especially the *LjCYC*2. In monocot, *RETARDED PALEA1* (*REP1*) controls palea development and floral zygomorphy in rice, whose flower is different from that of *A*. *majus* or *L*. *japonicus*. In our *C*. *faberi* transcriptome data, we identified 35 *TCP* genes, of which 10 *CIN*-like genes and one *PCF*-like gene showed significantly higher expression levels in the vegetative buds compared to the flower buds. qRT-PCR analysis revealed that the expression level of *BRC2* was higher in the flower buds than the vegetative buds (Fig. 8e), suggesting that it might play important role in the regulation of floral zygomorphy of *C*. *faberi*.

### Genes related to the regulation of flowering

Previous studies showed that the flowering of *Arabidopsis* was regulated by four pathways, including autonomous pathway, vernalization pathway, gibberellic acid (GA)-dependent pathway and the photoperiod pathway. And *CCA1* influenced the circadian rhythm, overexpression of *CCA1* resulted in long hypocotyls and late flowering (Wang and Tobing 1998). *CO* involved in the photoperiod pathway in *Arabidopsis* and acted as a floral promoter, which was regulated by the circadian clock (Yano et al. 2000). The temporal and spatial regulation of *CO* turned out to be important to the photoperiod-dependent induction of flowering (An et al. 2004). Early in a day, the expression of *CO* was low, then increased rapidly 10 h after dawn and peaked at around 15 h (Suárez-López et al. 2001). *LFY* is a major floral meristem regulator, its overexpression caused early flowering in transgenic *Arabidopsis* (Benlloch et al. 2007). A single *PHYA* gene, *LHY* gene and three *CCA1* genes involved in the photoperiod pathways showed more highly expressed in the vegetative buds than in the flower buds. Two *VRN* genes playing important roles in the vernalization pathway were detected in our study, and were found to be more highly expressed in the vegetative buds than in the flower buds. Besides, DEGs related to the autonomous pathway were also detected, including two *FCA* genes, a *FLD* gene and a *FY* gene. qRT-PCR revealed that expression of *LFY* was higher in the vegetative buds and expression of *CO*, *SOC1*, *CCA1* was higher in the flower buds, which was consistent with the RNA-Seq data (Fig. 8g–j). Expression of *CCA1* was significantly higher in the flower buds than the vegetative buds. This observation suggesting that the mechanism of flowering regulation in *C*. *faberi* might be similar to *Arabidopsis*.

In conclusion, the current RNA-seq and DEG analysis revealed 393 genes associated to the floral development, including 35 TCP transcription factors (TFs), 118 MADS-box genes and 240 flowering time related genes. A total of 173 DEGs were identified, including 12 TCP genes, 34 MADS-box genes and 28 flowering time related genes. The transcriptome database in the present study will be a valuable supplement to the genomic sequence dataset of *C*. *faberi*, and will serve as an important public information platform for further studies on the flower development mechanism in *C*. *faberi*.

## Additional files

**Additional file 1: Table S1.** Gene-specific primers for qRT-PCR.

**Additional file 2: Fig. S1.** Length distribution of unigenes.

**Additional file 3: Table S2.** Result of metabolic pathway analysis for unigenes by KEGG annotation.

**Additional file 4: Table S3.** Unigenes that share homology with MADS-box genes.

**Additional file 5: Table S4.** Unigenes that share homology with TCP family genes in *C*. *faberi*.

**Additional file 6: Table S5.** Unigenes that related to the flowering time in *C*. *faberi*.

**Additional file 7: Table S6.** The functional annotation for the DEGs related with flower development in *C*. *faberi*.

**Additional file 8: Table S7.** DEGs related to the flowering time in *C*. *faberi*.
