# Supplementary material for: De novo transcriptome sequencing and comparative analysis to discover genes related to floral development in Cymbidium faberi Rolfe
Source: Springerplus. 2016 Aug 30;5(1):1458. doi: 10.1186/s40064-016-3089-1 (PMC5082062; doi:10.1186/s40064-016-3089-1)
Supplement: Supplementary file 1 — Additional file 1: Table S1. Gene-specific primers for qRT-PCR. [file 40064_2016_3089_MOESM1_ESM.doc]

**Table S1 Gene-specific primers for qRT-PCR**

| unigenes | primer name | primer sequence |
| --- | --- | --- |
| 046750(*BRC2*) | HF | 5′-CAGTTCAATGGCTGCTCAAT-3′ |
|  | HR | 5′-GCTCTTCGCACTCTGAAGC-3′ |
| 047433(*TCP4*) | T4F | 5′-ATTTGGCCAAGGTCAGATTC-3′ |
|  | T4R | 5′-ATATGAACCCGAGGAACGAG-3′ |
| 027439(*PI*) | PIF | 5′-AAACGCTCCAGAATGGTCTT-3′ |
|  | PIR | 5′-ACCCGATGTCCAGTTCTCTC-3′ |
| 035630(*AP1*) | APF | 5′-ATCCTCAGGAAGACTTGCGT-3′ |
|  | APR | 5′-GTTTCAAGCTGCTGCTCAAG-3′ |
| 052055(*DEF*) | DEFF | 5′-AAATCAAGGAACTGCGTGGT-3′ |
|  | DEFR | 5′-TCCTGTGGGTTTCTTGAGAGT-3′ |
| 061575(*AG*) | AGF | 5′-GTGGCTAGTCGGACAAGGAT-3′ |
|  | AGR | 5′-CACCTTCTGAACGGCTACCT-3′ |
| 067186(*SOC1*) | SOC1F | 5′-CCATTGAGCGTTACAGGATG-3′ |
|  | SOC1R | 5′-CAACCTGTTCTTCCAGCAAA-3′ |
| 067695(*CO*) | COF | 5′-GAGAGATCGGTGATGATGGA-3′ |
|  | COR | 5′-CGTATCGTCTTCTCAAACCG-3′ |
| 061831(*LFY*) | LFYF | 5′-CAGTGCCATGAGTTTCTGCT-3′ |
|  | LFYR | 5′-GCGTACCGGAATACAAGGTT-3′ |
| 073329(*CCA1*) | CCA1F | 5′-AGAAGTGGACGGATGAGGAG-3′ |
|  | CCA1R | 5′-GTCATGCCTTCATTCCCTTT-3′ |
| JX560732(*CfGAPDH*) | CF | 5′-GTCAACGATCCGTTCATCAC-3′ |
|  | CR | 5′-GGTTCCTGATGCCAAAGACT-3′ |
