# Supplementary figures and images for: De novo transcriptome sequencing and comparative analysis to discover genes related to floral development in Cymbidium faberi Rolfe
Source: Springerplus. 2016 Aug 30;5(1):1458. doi: 10.1186/s40064-016-3089-1 (PMC5082062; doi:10.1186/s40064-016-3089-1)

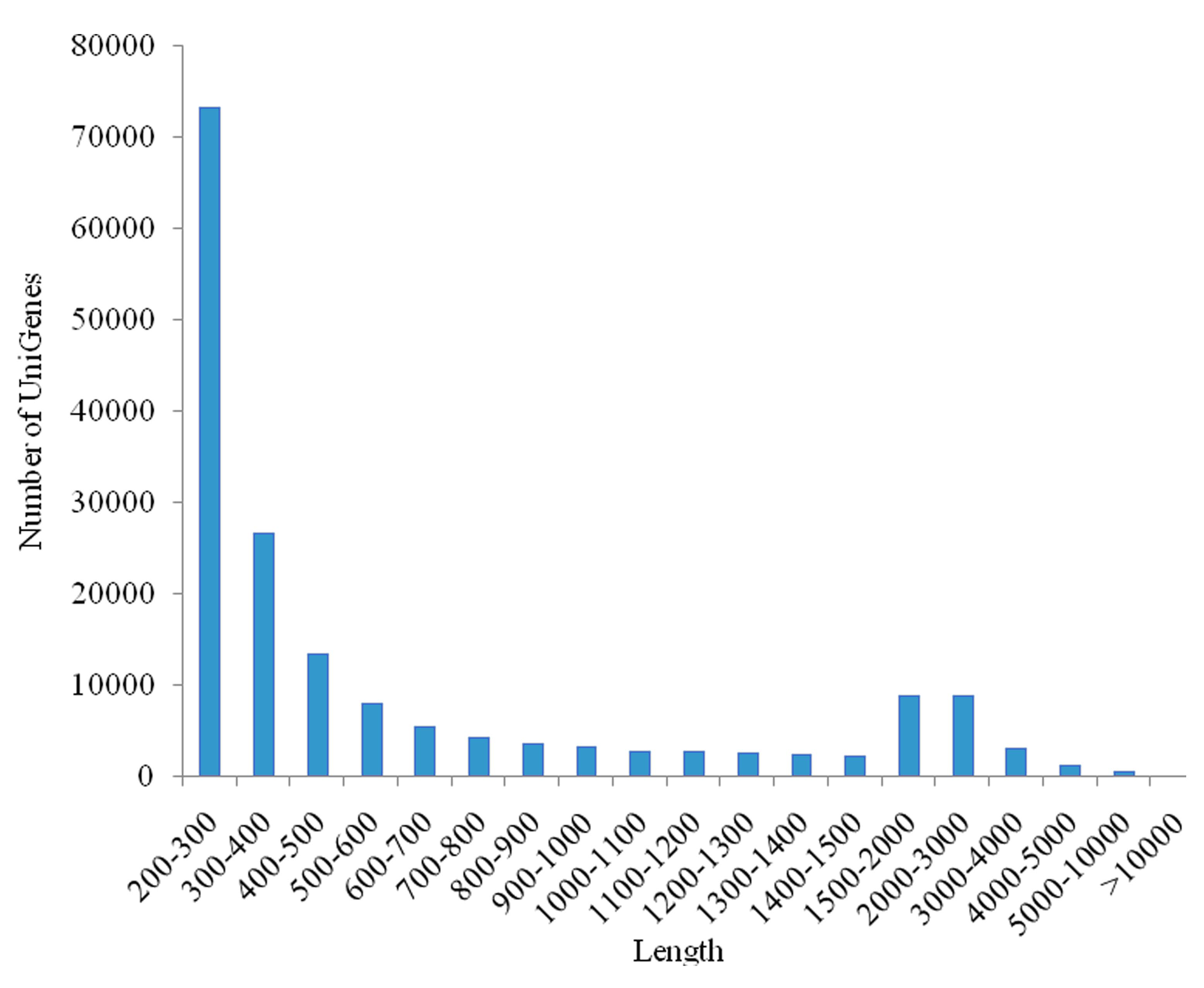

Supplement: Supplementary file 2 — Additional file 2: Fig. S1. Length distribution of unigenes. [file 40064_2016_3089_MOESM2_ESM.tif]
