# Supplementary material for: De novo transcriptome sequencing and comparative analysis to discover genes related to floral development in Cymbidium faberi Rolfe
Source: Springerplus. 2016 Aug 30;5(1):1458. doi: 10.1186/s40064-016-3089-1 (PMC5082062; doi:10.1186/s40064-016-3089-1)
Supplement: Supplementary file 5 — Additional file 5: Table S4. Unigenes that share homology with TCP family genes in C. faberi. [file 40064_2016_3089_MOESM5_ESM.doc]

**Table S4 Unigenes that share homology with TCP family genes in *C*. *faberi***

| Category | Gene ID | Homologous gene |
| --- | --- | --- |
| PCF | Unigene004959 | *TCP7* |
|  | Unigene032551 |  |
|  | Unigene055475 |  |
|  | Unigene057095 |  |
|  | Unigene063655 |  |
|  | Unigene073797 | *TCP8* |
|  | Unigene028462 | *TCP9* |
|  | Unigene060855 |  |
|  | Unigene073983 |  |
|  | Unigene001204 | *PTF(TCP13)* |
|  | Unigene065297 |  |
|  | Unigene071040 |  |
|  | Unigene031770 | *TCP14* |
|  | Unigene028193 | *TCP15* |
|  | Unigene044849 |  |
|  | Unigene051363 | *TCP20* |
|  | Unigene051981 |  |
|  | Unigene055563 |  |
|  | Unigene062970 |  |
|  | Unigene045955 | *TCP23* |
| CYC/TB1 | Unigene046750 | *BRC2(TCP12)* |
| CIN | Unigene001005 | *TCP2* |
|  | Unigene005157 |  |
|  | Unigene083295 |  |
|  | Unigene111740 |  |
|  | Unigene051001 | *TCP3* |
|  | Unigene051002 |  |
|  | Unigene051003 |  |
|  | Unigene065991 |  |
|  | Unigene099391 |  |
|  | Unigene028588 | *TCP4* |
|  | Unigene047433 |  |
|  | Unigene151805 |  |
|  | Unigene069346 | *TCP24* |
|  | Unigene069347 |  |
